# Inequalities in Childhood Healthcare Access Among Racial and Ethnic Groups of Sub-Saharan Africa: A Narrative Review

**DOI:** 10.3390/children13030435

**Published:** 2026-03-23

**Authors:** Syed Hanzila Azhar, Andrea Sárváry, Attila Sárváry

**Affiliations:** 1 Institute of Health Sciences, Faculty of Health Sciences, University of Debrecen, Kassai u. 26, 4032 Debrecen, Hungary; syedhanzilaazhar@gmail.com; 2 Department of Psychology, Institute of Social Sciences, Faculty of Health Sciences, University of Debrecen, Sóstói u. 2-4., 4400 Nyíregyháza, Hungary; sarvary.andrea@etk.unideb.hu; 3 Department of Nursing and Integrative Health Sciences, Institute of Health Sciences, Faculty of Health Sciences, University of Debrecen, Sóstói u. 2-4., 4400 Nyíregyháza, Hungary

**Keywords:** health inequalities, racial and ethnic disparities, Sub-Saharan Africa, child mortality, child healthcare access, narrative review

## Abstract

**Background/Objective:** Child health serves as a foundational part of human development. Inequities in access to key health services remain high in Sub-Saharan Africa (SSA), most notably among children from disadvantaged, racially, or ethnically marginalized groups. The objective of this structured narrative review is to evaluate and aggregate the available evidence on racial/ethnic disparities in childhood healthcare access in SSA. **Methods:** A comprehensive search on African Index Medicus (AIM), Web of Science and PubMed for studies published between 2010 and 2025 was executed using relevant MeSH terms and Boolean operators. Studies on healthcare access inequalities among racial or ethnic groups in SSA were included. This study was conducted following Preferred Reporting Items for Systematic Reviews and Meta-Analyses (PRISMA) guidelines. **Results:** Ten articles were included. Ethnicity was an independent contributor to inequities in childhood healthcare across four domains: vaccination coverage (lower for Hausa/Fulani in Nigeria and Somali/Luhya in Kenya compared to dominant groups), timeliness of vaccination, child mortality (higher in economically and ethnically marginalized groups), and nutritional status (elevated stunting and underweight odds in certain ethnic minorities). **Conclusions:** Racial and ethnic inequalities in child healthcare access across SSA are driven by multi-factor structural, geographical, and cultural barriers. Although socioeconomic improvement reduces some disparities, it does not eradicate them, highlighting that ethnic identity continues to shape health outcomes independently. Addressing these disparities requires strengthening culturally inclusive healthcare delivery, improving access in underserved regions, and integrating ethnicity-disaggregated monitoring into national health systems.

## 1. Introduction

Early childhood healthcare is central to survival, growth, and long-term health outcomes [1]. Undernutrition during early life, particularly within the first 1000 days, is associated with impaired physical and cognitive development and increased morbidity and mortality [2,3]. The importance of child health is universally acknowledged, from reducing mortality to improving outcomes throughout their lives [4]. Child health inequalities are global and strikingly evident in Sub-Saharan Africa (SSA). Mortality rates in children under five years of age have fallen (from 93 per 1000 births in 1990 to 37 in 2022), while SSA retains a disproportionate share of the burden. In 2022, nearly 53% of the world’s 4.9 million deaths of children under five occurred in SSA [5]. Major contributors to child mortality in the region include malnutrition, inadequate immunization coverage, and limited access to essential primary healthcare services [6]. It is estimated that nearly two-thirds of child deaths in SSA could be prevented through effective and accessible health interventions [7]. Malnutrition in SSA is a central impediment. According to UNICEF, in Mali about 26% of children are stunted and around 2% have severe acute malnutrition (SAM). These nutrition-related gaps affect immune development and cognitive development [7]. Access barriers worsen the situation, as many rural impoverished families do not have clinics in close proximity, cannot afford care, or face other barriers. For example, many children miss routine vaccinations and follow-up care for pneumonia and diarrhea [8]. Access to healthcare in SSA is shaped by multiple interrelated dimensions, including geographic, financial, and social cultural factors [9]. Even where services are available, cost barriers, language differences, and cultural mismatches between providers and communities can prevent effective utilization, leaving children without adequate care [10]. SSA is one of the most ethnically and linguistically diverse regions globally, comprising 49 countries and hundreds of ethnic groups and languages [11]. This diversity is also reflected in religious composition, with Christianity and Islam remaining the predominant faiths, though their relative proportions are projected to shift over time [12]. This diversity exists within a complex socio-political context shaped by colonial-era borders that often divided ethnic groups across modern nation states, contributing to enduring structural and social inequalities that influence access to services (in the cases of the Somali nation cut off across Somalia, Ethiopia, Djibouti and Kenya and the Afar nation struggling between Ethiopia, Eritrea, and Djibouti) [13]. In some settings, notably South Africa, apartheid-era racial classifications continue to influence health system organizations and access to healthcare [14]. It is important to distinguish between the two key terms. Ethnicity refers to a shared cultural identity rooted in common ancestry, language, religion and tradition. It is a socially constructed category that is largely self-identified and shaped by local context. Race, on the other hand, is a socio-political construct used to classify populations based on perceived physical characteristics, most notably institutionalized through apartheid-era classifications in South Africa. Overall, ethnicity is deeply entrenched in social life (with common history, language and culture). Some intend to emphasize that these SSA ethnic differences “may sometimes result in divisions or violent conflict,” yet most of the time groups coexist harmoniously [15]. These social divides play a role in health: discrimination toward minority groups may influence not only health-seeking behavior but also health system responses. Across most of SSA these dynamics are primarily ethnic in nature, though in South Africa racial divisions continue to shape health access in ways that are distinct from the rest of the region. Empirical evidence demonstrates that ethnicity and, to a lesser extent, race are associated with disparities in access to child healthcare services in SSA. In Nigeria, significant differences in childhood immunization coverage have been documented across ethnic groups, with Hausa/Fulani children consistently exhibiting lower coverage compared to Igbo and Yoruba children, even after controlling for socioeconomic factors [16]. Similarly, in South Africa, stark racial disparities persist in healthcare access, with a substantially higher proportion of the White population covered by private health insurance compared to Black proportions, who rely predominantly on under-resourced public services [17]. These discrepancies are echoed in children: for instance, the under-five mortality rate among Black children remains much above that of White children, and immunization and nutrition indices lag for disadvantaged ethnic groups [18]. Ethnic minority and racially marginalized children often face intersecting disadvantages, including poverty, social exclusion, and discrimination, which collectively restrict access to essential health services [19].

Despite extensive literature documenting socioeconomic inequalities in child health across SSA, ethnic and racial disparities have received comparatively limited systematic attention. Most inequality studies focus on wealth, education, or geographic location, while ethnicity is less frequently analyzed, despite its inclusion in frameworks such as PROGRESS-Plus (“Place of residence, Race/ethnicity, Occupation, Gender, Religion, Education, Socioeconomic status, Social capital, plus others”) [19,20]. Although some national and cross-country analyses have identified ethnic differences in child mortality and service utilization, comprehensive multi-country evaluations remain scarce [19]. Qualitative studies further suggest that cultural beliefs, language barriers, and historic mistrust of health systems may hinder healthcare access for certain ethnic groups, though such findings are often context-specific and fragmented [21].

Despite the growing body of literature on socioeconomic inequalities in child health across SSA, ethnicity as an independent predictor of disparities in childhood healthcare access has received comparatively little systematic attention. Beyond vaccination coverage, relatively little is known about whether ethnic disparities extend to how often children receive vaccines, whether nutritional status follows similar patterns of inequality, and how factors such as armed conflict and population movement interact with ethnicity to further limit healthcare access. A comprehensive multi-country synthesis addressing these questions does not currently exist. Analysis using large-scale survey data, such as the Demographic and Health Survey (DHS), have demonstrated strong gradients in child health outcomes by wealth and education but have been constrained in examining ethnicity due to data limitations [22]. Given global commitments to the Sustainable Development Goals and the principle of “leaving no one behind”, the lack of ethnic disaggregated evidence represents a critical limitation in efforts to achieve equitable child health outcomes.

The aim of this structured narrative review is to synthesize evidence on racial and ethnic disparities in access to child healthcare services in SSA, examining the extent of these disparities across key indicators and identifying gaps related to ethnicity disaggregated data. Although both terms are retained in the title and aim to reflect the original search scope, the review findings are predominantly drawn from studies examining ethnic disparities, as racial disaggregation remains limited in the primary literature across most of SSA. The findings of this research can contribute to equitable child health policy and future research.

## 2. Materials and Methods

### 2.1. Search Strategy

A comprehensive literature search was performed according to PRISMA (Preferred Reporting Items for Systematic Reviews and Meta-Analyses) guidelines [23] using the African Index Medicus (AIM), Web of Science and PubMed databases for studies published between 2010 and 2025. The search strategy utilized a combination of Medical Subject Headings (MeSH) and several other keywords, connected via Boolean operators (AND, OR) to cover the breadth of the topic. The search strategy included the following keywords: “Childhood healthcare access,” OR “pediatric healthcare access,” OR “child health services,” AND “inequalities,” OR “disparities,” OR “barriers,” OR “inequities,” AND “racial groups,” OR “ethnic groups,” OR “minority populations,” AND “Sub-Saharan Africa,” OR “Sub-Saharan African countries.” A complete search strings for each database is provided as Supplementary Material S1. (Supplementary SA). A Boolean logic framework was employed to encompass all relevant variations of these concepts in order to ensure that a variety of studies examining disparities in healthcare access for children living in Sub-Saharan Africa were included. The search was limited to publications in the English language and free full-text versions of the publications. We acknowledge that restricting the search to free full-text and English-language articles may introduce selection bias; relevant studies from Francophone and Lusophone Africa may have been missed.

### 2.2. Eligibility Criteria

The studies were involved if they met the next inclusion and exclusion criteria.

#### 2.2.1. Inclusion Criteria

Studies were included in the review if they met the following criteria:

1. Focused on children within SSA.
2. Examined healthcare access inequalities among racial or ethnic groups.
3. Published between 2010 and 2025.
4. Written in the English language.

#### 2.2.2. Exclusion Criteria

Studies were excluded if they met the following criteria:

1. Did not focus on childhood healthcare access.
2. Addressed general healthcare access without race or ethnicity-specific analysis.
3. Were conducted outside SSA.
4. Were non-English publications.

### 2.3. Study Selection

**Figure 1:**
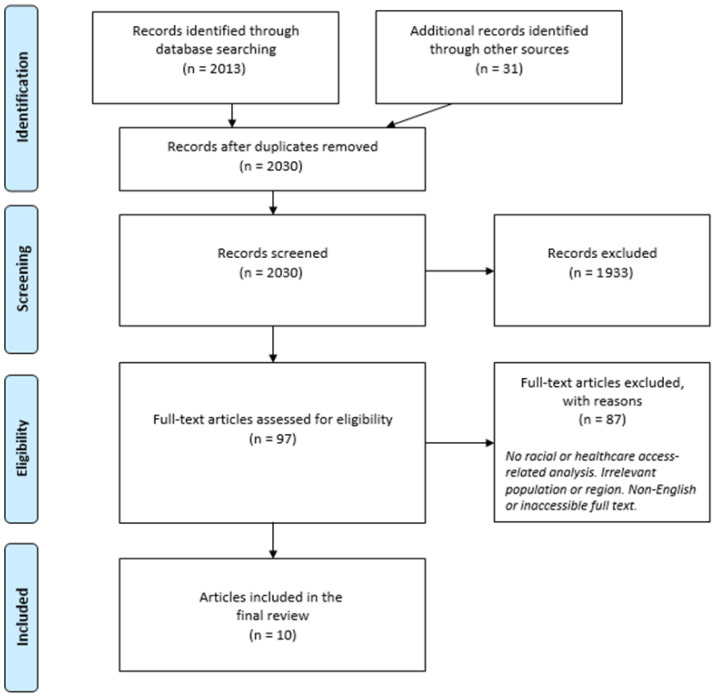
PRISMA flow diagram of study selection process.

The original database search yielded 2013 records, and a further 31 records were collected from additional sources, namely the reference lists of relevant articles and systematic reviews identified during full-text screening. After removing duplicates, 2030 records remained. Titles and abstracts were screened independently by two reviewers, leading to the exclusion of 1933 studies that did not meet inclusion criteria. Any discrepancies between the two reviewers were resolved through discussion and consensus. We then assessed the full texts of 97 articles for eligibility, excluding 87 (based on lack of racial/ethnic focus, irrelevant populations, or its inability to provide full text). Thus, 10 studies were included in this narrative review. No automation tools were used during screening process. The study selection process was guided by the PRISMA guidelines as illustrated in Figure 1 below.

### 2.4. Data Extraction and Synthesis

Data was independently extracted by both reviewers using a standardized extraction approach, including study design, country and setting, population characteristics, age range of population/children, racial or ethnic groups examined, and key findings related to healthcare access inequalities. Any kind of discrepancies were resolved through discussion and consensus.

A formal risk-of-bias assessment was conducted by using the JBI criteria for analytical cross-sectional studies. A narrative quality appraisal was performed for each included study, evaluating representativeness of the study sample, validity of outcome measurement, and appropriateness of statistical analyses. Studies were classified as having low, moderate, or high methodological concerns, shown in Supplementary Material S1; Supplementary SB as Table S1. The majority of included studies relied on nationally representative survey data (DHS or MICS), which lends methodological consistency, although cross-sectional designs limit causal inference. Quantitative effect measures were not pooled as the extracted data were synthesized narratively, focusing on reported patterns. Results were presented using a summary table and narrative descriptions. Formal assessment of heterogeneity, sensitivity analyses, and certainty-of-evidence grading were not performed. Gray literature was not systematically searched, which may introduce publication bias. The full database search strings are provided in Supplementary Material S1, Supplementary SB; the PRISMA 2020 checklist is provided in Supplementary Material S2.

## 3. Results

Across the ten included studies, several consistent patterns emerged regardless of country or study design. Children from ethnic minority groups were persistently less likely to be fully vaccinated and more likely to experience delays in receiving vaccines compared to children from dominant ethnic groups. Ethnic minority children also faced higher risks of malnutrition and elevated under-five mortality rates. These patterns remained even after accounting for socioeconomic and geographic factors, suggesting that ethnicity operates as an independent determinant of child health outcomes across SSA. The evidence base in this review largely concerns ethnic rather than racial disparities. This is not an omission on the part of the authors but rather a reflection of how data is reported in the primary literature across SSA, where ethnicity-disaggregated data is far more available than racial classification, the latter being most documented in South Africa. A total of 2013 records were identified through database searches and 31 from other sources. After reviewing 2030 records and assessing 97 full-text publications for eligibility, 87 were rejected given reasons, leaving 10 research that fulfilled all inclusion criteria. Eligible research was required to investigate ethnic or racial disparities in childhood healthcare access or outcomes among populations in SSA and to be published in English. The included research studies were carried out across Nigeria (n = 4), Kenya (n = 3), Ghana (n = 1), rural southern Guinea-Bissau (n = 1), and a pooled analysis encompassing 18 SSA countries (n = 1) (Table 1). Most employed DHS or Multiple Indicator Cluster Survey (MICS) data, while others relied on longitudinal monitoring or community-based cross-sectional surveys. Sample sizes varied from 382 children in urban informal settlements of Kenya to 138,312 children representing 45 ethnic groups in the multi-country DHS research. Collectively, these investigations evaluated how ethnicity effects access to and usage of childhood healthcare services. Across all study data, ethnicity appeared as a constant and independent driver of uneven child health outcomes, even after adjusting for socioeconomic and demographic characteristics. The data are arranged thematically into four core domains: ethnic disparities in vaccination coverage and completeness; timeliness of childhood vaccination; ethnic differences in child mortality; and anthropometric and nutritional inequalities among ethnic groups.

**Table 1:** Summary of studies’ characteristics and key findings.

Code	Author(s) & Year	Country	Study Design	Sample Size & Population	Study Aim	Key Findings Related to Ethnicity and Childhood Healthcare Access
S1	Afolabi RF et al. (2021) [24]	Nigeria	Cross-sectional analysis of 2018 Nigeria DHS; generalized linear mixed model	3980 children aged 12–23 months.	To explore determinants of complete vaccination among Hausa/Fulani, Igbo, and Yoruba groups.	Complete vaccination was lowest among Hausa/Fulani (18.2%) compared to Yoruba (40.8%) and Igbo (56.3%) children.
S2	Masters NB et al. (2019) [25]	Kenya	Cross-sectional; 2014 Kenya DHS; Multinomial Logistic Regression.	4052 children aged 12–23 months.	To examine sociodemographic predictors of vaccination, focusing on Somali children.	Somali children had much lower full vaccination (61.9%) than national average (79.4%) and higher odds of under- or non-vaccination compared to Kikuyu children.
S3	Ettarh RR et al. (2012) [26]	Nairobi, Kenya	Longitudinal surveillance data (2006–2010); Cox regression	2317 children aged 9–59 months.	To examine ethnic disparities in measles vaccination timeliness and coverage.	Luhya and minor ethnic groups were more likely to have delayed measles vaccination than Kikuyu children.
S4	Adokiya MN et al. (2017) [27]	Ghana	Cross-sectional cluster survey (2016); logistic regression.	600 children aged 12–23 months.	To evaluate immunization coverage and its associated factors.	Frafra children were significantly more likely to be under-immunized compared to Akan children (AOR = 4.71).
S5	Egondi T et al. (2015) [28]	Nairobi, Kenya	Cross-sectional; 2012 Nairobi Slum Survey; Concentration index.	382 children aged 12–23 months.	To determine the degree and determinants of immunization inequality among the urban poor.	Immunization inequality varied by ethnicity, concentrated among Luhya and Luo groups. Mother’s education explained 78% of this inequality.
S6	Antai D. (2011) [29]	Nigeria	Cross-sectional analysis of 2003 Nigeria DHS data; logistic regression.	6029 children <5 years born to 3725 mothers in 365 communities.	To examine the mediatory effects of ethnicity and socioeconomic position on under-five mortality.	Igbo and Yoruba children had significantly lower under-five mortality than Hausa/Fulani/Kanuri children, with ethnicity remaining independent after adjustment.
S7	Adedini SA et al. (2015) [30]	Nigeria	Cross-sectional; 2008 Nigeria DHS (1993–2008); Cox regression.	104,808 live births from 33,385 women aged 15–49.	To examine ethnic differentials in under-five mortality in Nigeria.	Yoruba and Igbo children had significantly lower under-five mortality compared to Hausa/Fulani/Kanuri children (HR = 0.39 and HR = 0.58 respectively).
S8	Ghose B & Yaya S. (2020) [31]	Nigeria	Cross-sectional; 2017 Nigeria MICS; Multivariable regression.	34,139 women aged 15–49.	To assess ethnic disparities in fertility (parity) and child mortality and their relationship.	Hausa women had the highest child death prevalence, with significantly higher odds remaining after adjustment compared to other ethnic groups.
S9	Fazzio I et al. (2011) [32]	Rural southern Guinea-Bissau	Retrospective; birth histories (1977–2007); Kaplan–Meier and Cox regression.	32,215 children born to 7854 women over 30 years.	To estimate child mortality trends and examine effects of ethnicity, historical events, and distance to health centers.	Ethnicity was strongly associated with child mortality, with Balanta and Fula children showing the highest mortality in different time periods.
S10	Tusting LS et al. (2023) [33]	18 countries in Sub-Saharan Africa	Cross-sectional analysis of 37 Demographic and Health Surveys (2006–2019)	138,312 children <5 years across 45 ethnic groups.	To assess how ethnicity is associated with anthropometric deficits (stunting, wasting, and underweight).	Hausa children had 32% higher odds of stunting (aOR = 1.32) and Igbo children had 69% lower odds (aOR = 0.31) compared to Fula children.

### 3.1. Ethnic Disparities in Vaccination Coverage and Completeness

Inequalities in vaccination coverage among ethnic groups were a frequent finding in the examined studies. In Nigeria, S1 [24] found significant ethnic heterogeneity in full vaccination among children aged 12–23 months. The rate of full vaccination was 56.3% among Igbo children, 40.8% among Yoruba, and only 18.2% among Hausa/Fulani (Table 2). After controlling for confounders, Igbo children had higher odds of being fully vaccinated (aOR = 1.38; 95% CI: 1.20–1.59) compared to the Hausa/Fulani. The effect of household wealth reinforced these variations, whereby higher wealth status was associated with an increased likelihood of full vaccination for all ethnic groups, but Hausa/Fulani were always the least covered (aOR = 1.65; 95% CI: 1.04–2.61) compared to Igbo (aOR = 2.55; 95% CI: 1.20–5.44) and Yoruba (aOR = 4.22; 95% CI: 1.27–13.96). This finding reflects the intertwined significance of ethnicity and socioeconomic status in shaping access to immunization in Nigeria. Similar patterns were described in Kenya whereby ethnic identification conditioned children’s odds of receiving basic vaccines. For example, S2 [25] reported that Somali children were less likely to receive full vaccine coverage compared to Kikuyu children (aOR = 0.41; 95% CI: 0.29–0.57). Even after adjusting for socioeconomic and regional factors, however, the effect of ethnicity persistently emerged as a structural barrier to equitable immunization coverage. Discrepancies could be corroborated by the Somali’s socio semi-nomadic lifestyle, marginalization within society, and lack of health infrastructure. These outcomes corroborate S3 [26] which demonstrated that in Nairobi’s informal settlements, versus Kikuyu children, Luhya children and other minority groups received the measles vaccine significantly later, reaffirming the different structural and social barriers to timely vaccination. In Ghana, differences were present, though less pronounced. S4 [27] observed a high full vaccination coverage of 89.5% in Techiman Municipality (Akan group) while other groups had lower coverage, which indicates persistent structured and social barriers to timely immunization.

**Table 2:** Ethnic disparities in childhood vaccination coverage and completeness across Nigeria, Kenya, and Ghana.

Country/Study		Ethnic Group	Coverage/Pattern	Effect Size	Interpretation
Nigeria (Afolabi et al., 2021) [24]		Igbo	56.3%	AOR 1.38 (1.20–1.59)	Higher coverage than Hausa/Fulani
		Yoruba	40.8%	—	Intermediate coverage
		Hausa/Fulani	18.2%	Reference	Lowest coverage
	Wealth Effect	Igbo (Highest Wealth)	—	AOR 2.55 (1.20–5.44)	Wealth improves coverage
		Yoruba (Highest Wealth)	—	AOR 4.22 (1.27–13.96)	Strongest wealth effect
		Hausa/Fulani (Highest Wealth)	—	AOR 1.65 (1.04–2.61)	Still lowest even with wealth
Kenya (Masters et al., 2019) [25]		Somali	Lower than Kikuyu	AOR 0.41 (0.29–0.57)	Ethnic & structural barriers
Kenya (Masters et al., 2019) [25]		Kikuyu	Higher coverage	Reference	Advantaged group
Kenya (Ettarh et al., 2012) [26]	Timeliness	Luhya + minorities	Delayed measles vaccination	RRR 0.65 vs. Kikuyu	Delayed access
		Kikuyu	More timely vaccination	Reference	Better access
Ghana (Adokiya et al., 2017) [27]		Akan	Overall coverage 89.5%	Reference	Majority group
		Frafra	Lower coverage	AOR 4.71 (1.46–15.18)	Higher odds of incomplete immunization
		Kusaasi	Higher coverage	AOR 0.09 (0.02–0.51)	Lower odds of incomplete immunization

Note: “—" implies that an adjusted effect size is not provided in the study for that specific comparison of ethnicity and outcome. The absence of a value reflects non-reporting, not a reflection of no association. AOR: adjusted odds ratio; RRR: Relative risk ratio refers to the likelihood of on-time vaccination for measles.

### 3.2. Timeliness of Childhood Vaccination Among Ethnic Groups

In addition to coverage and completion, other studies have examined ethnic differences in the timeliness of vaccination as an important factor in accessing healthcare (S3 [26] and S5 [28]). S3 [26] studied the timing of measles vaccination in the Korogocho slum of Nairobi and identified significant ethnic differences in vaccine delays. Cox regression analysis of 2317 children, in S3 [26], showed that children of Luhya and smaller minority ethnic groups were significantly more likely to experience delayed measles vaccination compared to Kikuyu children. The ethnic differences in vaccination remained inequities despite the children living in the same metropolitan area. S5 [28] provided corroborative evidence showing that ethnic minority households in informal settlements in Nairobi experienced structural barriers such as inequitable healthcare service provision and different understandings of illness and preventive health practices, which contributed to delayed vaccine uptake, even when individuals eventually finished their vaccination schedule. Across studies, delays were linked to a combination of sociocultural beliefs linked to ethnicity, maternal understanding of the immunization schedule, and availability of services from minority communities. The similarities in urban and rural settings in Kenya indicates that ethnicity constitutes both a cultural and structural barrier to timely access to healthcare.

### 3.3. Ethnic Differences in Child Mortality and Survival Outcomes

Ethnic variations encompass more than vaccine access, extending to essential child survival outcomes. Several studies based in Nigeria and Guinea-Bissau have identified large ethnic differentials in under-five mortality. S6 [29] observed that under-five mortality was significantly higher for children from Hausa/Fulani/Kanuri mothers than children from Yoruba mothers (high risk vs. low risk). Once sociodemographic covariates at both the individual and community levels were applied, the children of Igbo and other ethnic minority groups had significantly lower odds of mortality compared to children from Hausa/Fulani/Kanuri households. The majority of the reported differences in child mortality were largely attributed to differences in maternal education, health service utilization, and access to prenatal care among ethnic groups; however, ethnicity remained a significant correlate of child mortality above and beyond socioeconomic status, utilization of health, and education. Cultural and structural determinants in the context of ethnic identity appeared to be an important consideration. S7 [30] also found that Hausa/Fulani children were consistently more likely to die than the Yoruba or Igbo children. The authors attributed this to existing cultural beliefs associated with the previously mentioned practices (e.g., early marriages, larger family sizes, and reduced autonomy of women in decision-making regarding child health). S8 [31], using data indicators from a multiple-indicator cluster survey, concluded that after accounting for potential confounding variables (aOR = 1.43; 95% CI: 1.09–1.86), Hausa/Fulani mothers had more child death prevalence than mothers from other ethnic backgrounds. These studies establish a strong association between child mortality and ethnicity as an independent risk factor for child mortality even after taking socioeconomic, educational and regional variables into account. Evidence from Guinea-Bissau considerably extended the spatial and temporal range of these inequities. S9 [32] examined child mortality patterns between 1977 and 2007 and reported continuing ethnic inequalities, even though there was overall national progress. The Balanta group had the highest rates of under-five mortality in earlier years, while between 2002 and 2007, Fula infants were at the greatest risk. Even with polygyny and distance to health centers measured, ethnicity remained a significant predictor of mortality. The hazard of under-five mortality was 1.5 times higher in the period of civil war between 1997 and 2001 than in prior periods, indicating that ethnic vulnerability was intensified during moments of political instability.

### 3.4. Anthropometric and Nutritional Inequalities Among Ethnic Groups

Ethnic disparities in child health were seen in anthropometric and nutritional outcomes, indicating wider inequities in living conditions and access to healthcare and nutritional services (Table 3). S10 [33] performed a cross-sectional analysis of 37 DHS across 18 Sub-Saharan African nations, revealing significant ethnic disparities in stunting, wasting, and underweight among children under five years of age. In comparison to the Fula reference group, Hausa children had markedly elevated chances of stunting (aOR = 1.32; 95% CI: 1.21–1.44) and underweight (aOR = 1.13; 95% CI: 1.03–1.24). Conversely, Igbo children had significantly reduced risks of stunting (aOR = 0.31; 95% CI: 0.27–0.35) and underweight (aOR = 0.15; 95% CI: 0.08–0.29), indicating that ethnic identification profoundly affected nutritional status. The authors discovered that ethnic group affiliation had a more significant impact on anthropometric outcomes than wealth, sanitation, or maternal education, highlighting the severity of ethnic health disparities in the region (Table 3).

**Table 3:** Adjusted odds ratios for anthropometric deficits among ethnic groups.

Ethnic Group	Reference Group	Stunting (aOR, 95% CI)	Wasting (aOR, 95% CI)	Underweight (aOR, 95% CI)	Interpretation
Hausa	Fula	1.32 (1.21–1.44)	—(not highlighted; prevalence highest overall)	1.13 (1.03–1.24)	Hausa children have higher odds of stunting and underweight than Fula children.
Igbo	Fula	0.31 (0.27–0.35)	—(not highlighted)	0.15 (0.08–0.29)	Igbo children have substantially lower odds of stunting and underweight than Fula children.
Bamileke	Fula	—	0.13 (0.05–0.32)	0.15 (0.08–0.29)	Bamileke children have markedly lower odds of wasting and underweight.
Mandinka	Fula	—	0.87 (0.76–0.99)	—	Mandinka children have slightly lower odds of wasting.

Note: “—” implies that an adjusted effect size is not provided in the study for that specific comparison of ethnicity and outcome. The absence of a value reflects non-reporting, not a reflection of no association. aOR: adjusted odds ratio.

These findings align with national-level research, S1, and parallel evidence from Kenya and Nigeria demonstrating that ethnicity shapes care-seeking behaviors, maternal autonomy, and access to child health services, S2 and S3. In marginalized ethnic groups, limited dietary diversity, reduced maternal health literacy, and systematic barriers to nutrition-related services compound these risks.

### 3.5. Socioeconomic and Cultural Mediators of Ethnic Inequalities

Whilst socioeconomic variables frequently decreased ethnic inequalities in multivariate studies, they rarely abolished them, demonstrating that ethnicity functioned both independently and interactively with other determinants. Across studies, maternal education and household affluence consistently mediated the connection between ethnicity and child health outcomes. For example, S1 observed that maternal education and place of delivery explained part of the disparities between Hausa/Fulani and Igbo offspring; however, ethnic gaps persisted after correction. Similarly, S6 showed that access to physician-provided community prenatal care somewhat explained ethnic differentials in under-five mortality but did not entirely account for them. Cultural influences were also consistently highlighted as explanatory processes. Studies in Kenya and Nigeria emphasized how ethnic-specific attitudes about sickness, vaccination safety, and childrearing related to health behavior inequalities. For example, S3 observed that Luhya and Luo populations in Nairobi associated vaccination with illness, resulting in delayed uptake, while S1 stated that traditional norms within Hausa/Fulani ethnic groups prioritized home births and limited women’s healthcare decision-making power, contributing to low rates of vaccination.

Taken together, the evidence across all four domains points to a consistent picture of cumulative disadvantage among ethnic minority children in SSA. Disparities in vaccination coverage and timeliness, child mortality and nutritional status do not occur in isolation but tend to cluster within the same marginalized ethnic populations, suggesting shared underlying structural and cultural barriers rather than outcome-specific causes.

## 4. Discussion

This narrative review analyzed ten papers that explored ethnic and racial differences in childhood healthcare access and outcomes across SSA. The aggregate evidence suggests that ethnicity remains a primary and independent factor of inequitable child health outcomes, with disparities persisting even after adjusting for socioeconomic and geographic factors, demonstrating that ethnicity, and in certain contexts, race continue to impact the distribution of health chances through both structural and cultural mechanisms.

Across the main outcome domains examined, ethnic disparities in vaccination coverage appeared the most consistent and well-documented finding, reported across multiple countries and study designs. Disparities in child mortality were also clearly evidenced but showed greater variation across contexts, being most pronounced where ethnicity intersected with geographic isolation or political marginalization. Nutritional disparities, while significant where examined, were less frequently reported, reflecting the more limited number of studies addressing this domain. This pattern suggests that vaccination coverage may be the most immediate and measurable entry point for monitoring and addressing ethnic inequalities in child health across SSA.

### 4.1. Ethnic Disparities in Immunization Coverage

In Nigeria, it was observed that Hausa/Fulani children consistently showed lower vaccination coverage than southern ethnic groups [24]. Even after correcting for maternal education, wealth, and geography, Hausa/Fulani children remained considerably less likely to complete vaccination schedules, suggesting that ethnic identification operates beyond socioeconomic status as a driver of immunization. Similar tendencies were observed in Kenya, where studies reported that children belonging to Somali, Luhya, and Luo ethnic groups had considerably lower vaccination completion rates than those from the Kikuyu majority [25,26,28]. These findings illustrate how ethnicity combines with geography, income, and societal views to restrict access to preventative health. Marginalized ethnic groups generally occupy remote or conflict-prone locations where health infrastructure is poor and outreach services are sporadic [34]. For example, nomadic and semi-nomadic communities such as the Somali in Kenya and the Hausa/Fulani in northern Nigeria frequently migrate, making it difficult to maintain routine immunization schedules [24,25]. Collectively, these studies affirm that ethnicity acts not only as a proxy for poverty or distance but also as a sign of past marginalization and suspicion toward state health institutions.

These structural barriers do not affect all ethnic minorities in the same way. Pastoralist and semi-nomadic communities such as the Somali in Kenya and the Fulani in Nigeria face barriers that are primarily logistical in nature, as frequent movement makes it difficult to maintain routine vaccination schedules and access facility-based care. Sedentary ethnic minorities in politically marginalized regions, such as those in northern Nigeria or rural Guinea-Bissau, face a different set of challenges rooted in political exclusion, historical neglect and chronic underinvestment in health infrastructure. The impact of conflict also varies considerably by context. In some settings it disrupts health services temporarily, while in others it reflects a deeper and more persistent pattern of ethnic marginalization by the state. Recognizing these distinctions matters because they point to the need for different policy responses depending on the specific barriers faced by each group.

### 4.2. Vaccination Timeliness

Beyond overall coverage, the timeliness of immunization emerges as another factor of disparity. Studies from Kenya demonstrated that ethnic minority children were more likely to undergo delayed immunization, notably for measles and DTP3 vaccines [25,26,28]. Ettarh RR et al. (2012) revealed that children from the Luhya and smaller minority groups in Nairobi’s informal settlements received measles immunizations much later than Kikuyu children, even when they eventually finished the program [26]. Such delays enhance vulnerability to epidemics of avoidable infections and represent deeper difficulties in continuity of care. Cultural considerations and service delivery limitations contribute to these delays. Some caregivers from minority groups experienced discomfort or fear of discrimination in government clinics, while language issues between providers and parents further impeded good communication and follow-up [25,26]. In addition, health campaigns typically fail to target nomadic or border-region people whose movement patterns do not correspond with static service models. Together, these criteria suggest that fair access is not only about whether children receive care but also when and how regularly they do so [35].

### 4.3. Ethnic Disparities in Child Mortality

Several studies in this study showed considerable ethnic disparities in child survival [29,30,31,32]. In Nigeria, children of Hausa/Fulani/Kanuri descent experienced considerably higher under-five mortality than Yoruba or Igbo children, even after controlling for maternal education, wealth, and place of residence. Similarly, Hausa women displayed both increased fertility and a higher frequency of child fatalities compared with other ethnic groups [31]. These discrepancies show that structural inequities such as poor healthcare infrastructure, decreased maternal health service utilization, and sociocultural barriers intersect with ethnicity to determine survival outcomes [36,37]. Beyond Nigeria, it was observed in Guinea-Bissau that child mortality varied sharply by ethnic group and fluctuated between political periods [32]. The Balanta and Fula youngsters continuously demonstrated greater mortality rates than Beafada and other minority ethnic communities, notably during the civil war period when ethnic polarization and instability hindered access to healthcare. This research indicates that ethnic inequalities are not static but evolve in response to political and social forces. Ethnicity thus intersects with governance, conflict, and power relations to determine which people are protected by or excluded from healthcare [38,39].

### 4.4. Nutritional and Anthropometric Inequalities

Nutritional differences by ethnicity were also noticeable. Drawing on data from 18 SSA nations, it demonstrated that children from marginalized ethnic groups, particularly the Hausa, Fula, and other minorities, had significantly greater odds of stunting and underweight compared to the majority groups [33]. These discrepancies persisted despite correcting for socioeconomic characteristics, demonstrating that ethnicity independently influences nutritional outcomes. Cultural feeding traditions, food instability, and unequal access to nutrition programs all contribute to these trends [40].

In Nigeria, similar inequalities were identified between the Hausa/Fulani and southern ethnic groups, while in Kenya, children from Somali and Luhya villages had lower anthropometric indices than Kikuyu children [24,29]. These findings show that health inequities in SSA extend beyond service usage to incorporate the broader social determinants of health. Nutritional outcomes are determined by the interplay of culture, livelihood, and environmental context dimensions directly related to ethnic identity and geographic location [41].

### 4.5. Socioeconomic and Cultural Mediators

Although financial status, maternal education, and urban location considerably improve child health, these factors only partially explain ethnic and, where documented, racial discrepancies. It has found that even after controlling for socioeconomic factors, ethnic differentials in child mortality and vaccine coverage persisted [24,29]. This persistence shows that systemic racism, discrimination, and cultural mismatch between service providers and ethnic minority users play key roles [42]. Qualitative evidence across included studies further illuminates these pathways [25,26]. Many caregivers from minority groups mentioned experiences of stigma or exclusion in public clinics, while others noted culturally insensitive practices or language hurdles that impeded care-seeking. Patriarchal gender norms within some ethnic groups, such as the Hausa/Fulani, were also connected with delayed care decisions, since mothers often lacked autonomy to seek medical help for their children [24]. These variables demonstrate that tackling inequality needs not just enhancing service coverage but also reforming the institutional and cultural conditions that support exclusion [43].

Across the ten studies, numerous consistent trends emerge. First, ethnicity exerts an independent influence on childhood healthcare access and outcomes across many nations and datasets. Second, these discrepancies remain even in countries with relatively high total health service coverage, suggesting that aggregate advances can disguise underlying inequities. Third, the magnitude of difference varies depending on context, being most severe where ethnicity intersects with geographic marginalization (northern Nigeria, northeastern Kenya, and rural Guinea-Bissau). Cumulative evidence supports the notion that ethnic and racial differences are rooted in both structural and societal processes, though the racial dimension is most clearly documented in the South African context. Structurally, ethnic minorities generally locate in places with limited healthcare infrastructure, fewer skilled personnel, and greater transportation expenses. Sociocultural, historical discrimination, linguistic hurdles, and divergent health attitudes restrict utilization even when resources are available. Thus, ethnicity acts as an axis of cumulative disadvantage reinforced by location, class, and cultural distance from state institutions.

### 4.6. Policy and Programmatic Implications

The main findings of this analysis have substantial implications for health policy and programming in SSA. To get toward the Sustainable Development Goal of universal health coverage, governments must integrate ethnicity-disaggregated monitoring inside national health information systems. Without such data, disparities remain undetected and ignored. Programs targeting immunization, nutrition, and child survival should involve culturally responsive approaches such as employing community health workers from minority groups, translating materials into local languages, and cooperating with traditional leaders to create trust. Promising approaches in this direction have been documented in the region, including community-based outreach programs that engage workers from within minority communities and targeted vaccination campaigns designed for mobile and nomadic populations [35]. Scaling such models and embedding them within national health systems represents a key priority for reducing ethnic disparities in child health.

Moreover, policy frameworks should realize that increasing infrastructure alone is insufficient. Efforts must simultaneously address the sociopolitical underpinnings of marginalization, including prejudice and regional neglect. Investments in health facilities in minority-dominated communities, combined with social protection measures and community-based outreach, can help decrease inequalities. Development partners and NGOs should also promote inclusive approaches that incorporate local ethnic communities in the design, delivery, and assessment of child health initiatives.

### 4.7. Knowledge Gaps and Future Research

Despite rising attention to equity, large knowledge gaps continue. Most research relied on secondary DHS or MICS data, which rarely provide nuanced measurements of ethnicity, prejudice, or cultural behaviors. Ethnicity categories are often inconsistently defined between countries, restricting comparability. Moreover, very few studies adopted longitudinal designs capable of capturing causal connections between ethnicity and health outcomes. There is also a conspicuous paucity of qualitative and mixed-methods studies that explore the daily experiences of oppressed ethnic groups. Future systematic reviews on this topic should explicitly incorporate qualitative and mixed-methods evidence to complement the quantitative patterns identified here, as such studies would provide deeper insight into the cultural and experiential mechanisms that drive ethnic disparities in childhood healthcare access. Integrating community-level qualitative observations with large-scale quantitative analysis would provide a clearer understanding of how structural and cultural elements simultaneously cause disparities. Additionally, expanding data disaggregation in DHS and MICS surveys would allow for more detailed tracking of progress toward equity goals.

### 4.8. Limitations of the Review

This review has several important limitations that should be considered when interpreting the findings. First, the review was not prospectively registered in PROSPERO, which constrains the verifiability of pre-specified methods. Second, the possibility of publication bias cannot be excluded; studies reporting significant ethnic disparities may be more likely to be published, potentially overstating the magnitude of inequalities. Third, the review is restricted to published, English-language studies, potentially eliminating significant research from French- and Portuguese-speaking African countries. Fourth, the variability of outcome measures among studies ranging from vaccine coverage to mortality and nutrition limited quantitative synthesis. Fifth, most included investigations relied on cross-sectional data, which prevents causal inference. Finally, the absence of longitudinal studies in the evidence base prevents temporal inference about whether these disparities are widening or narrowing over time.

## 5. Conclusions

The central contribution of this narrative review is to demonstrate, for the first time across multiple health domains and countries, that ethnicity operates as an independent and persistent determinant of child health inequity in SSA, a finding that calls on policymakers to urgently mandate ethnicity-disaggregated data collection within national health monitoring systems as a foundational step toward closing these gaps.

This research provides persuasive evidence that racial and ethnic inequities are persistent in childhood healthcare access across SSA. Children from marginalized ethnic groups are regularly less likely to be fully immunized, more likely to have delays in immunization, and face higher risks of malnutrition and mortality compared to their counterparts from dominant groups. These discrepancies continue even after accounting for socioeconomic and regional variations, indicating that ethnicity works as an independent predictor of injustice. Addressing these discrepancies involves more than expanding coverage; it necessitates culturally inclusive health systems, targeted investments in underserved places, and continuous efforts to reduce structural impediments. Incorporating ethnicity into national monitoring frameworks and policy formulation is not just an issue of social justice but a necessity for achieving equitable and universal child health coverage in the region.

## Data Availability

No new data was generated in this review.

## Supplementary Materials

The following supporting information can be downloaded at: https://www.mdpi.com/article/10.3390/children13030435/s1.
