# Supplementary material for: Inequalities in Childhood Healthcare Access Among Racial and Ethnic Groups of Sub-Saharan Africa: A Narrative Review
Source: Children (Basel). 2026 Mar 23;13(3):435. doi: 10.3390/children13030435 (PMC13025879; doi:10.3390/children13030435)
Supplement: Supplementary file 1 [file children-13-00435-s001.zip › Supplementary Material S1.pdf]

Supplementary SA: Search Strings

Database 1: PubMed

(

("child"[MeSH Terms] OR "infant"[MeSH Terms] OR "children"[tiab] OR "child"[tiab]

OR "pediatric"[tiab] OR "paediatric"[tiab] OR "under-five"[tiab])

)

AND

(

("healthcare"[tiab] OR "health care"[tiab] OR "health services"[MeSH Terms]

OR "health service utilization"[tiab] OR "healthcare access"[tiab]

OR "immunization"[MeSH Terms] OR "vaccination"[MeSH Terms]

OR "child mortality"[tiab] OR "under-five mortality"[tiab]

OR "malnutrition"[MeSH Terms] OR "stunting"[tiab] OR "underweight"[tiab]

OR "nutrition"[MeSH Terms])

)

AND

(

("health status disparities"[MeSH Terms] OR "healthcare disparities"[MeSH Terms]

OR "inequalities"[tiab] OR "disparities"[tiab] OR "inequities"[tiab]

OR "barriers"[tiab] OR "inequality"[tiab])

)

AND

(

("ethnicity"[MeSH Terms] OR "ethnic groups"[MeSH Terms]

OR "minority groups"[MeSH Terms] OR "racial groups"[tiab]

OR "ethnic groups"[tiab] OR "minority populations"[tiab]

OR "race"[MeSH Terms] OR "ethnicity"[tiab])

)

AND

(

("Africa South of the Sahara"[MeSH Terms] OR "Sub-Saharan Africa"[tiab]

OR "Nigeria"[tiab] OR "Kenya"[tiab] OR "Ghana"[tiab]

OR "Guinea-Bissau"[tiab] OR "South Africa"[tiab])

)

Filters applied: Publication date 2010/01/01–2025/12/31; Language: English; Full text available.

Database 2: Web of Science (Core Collection)

TS=(

(child* OR infant* OR pediatric* OR paediatric* OR "under five" OR "under-five")

)

AND TS=(

("health care" OR healthcare OR "health service*" OR immuniz* OR vaccin*

OR "child mortality" OR "under-five mortality" OR malnutrition

OR stunting OR underweight OR nutrition*)

)

AND TS=(

(inequalit* OR disparit* OR inequit* OR barrier* OR "unequal access")

)

AND TS=(

(ethnic* OR ethnicity OR "ethnic group*" OR "minority population*"
OR "racial group*" OR race OR "indigenous population*")
)
AND TS=(
("Sub-Saharan Africa" OR "sub-Saharan Africa" OR Nigeria OR Kenya
OR Ghana OR "Guinea-Bissau" OR "South Africa" OR Ethiopia
OR Tanzania OR Uganda OR Senegal OR Cameroon)
)

Refined by: Publication Years: 2010–2025; Language: English.

Database 3: African Index Medicus (AIM)

(child OR children OR infant OR pediatric OR paediatric)

AND

(healthcare OR "health care" OR immunization OR vaccination
OR "child mortality" OR nutrition OR stunting)

AND

(inequality OR inequity OR disparity OR barrier)

AND

(ethnicity OR "ethnic group" OR race OR "minority population")

AND

("Sub-Saharan Africa" OR Africa)

Filters: 2010–2025; English language

Supplementary SB

Table S1. Quality Appraisal of Included Studies Using JBI Criteria for Cross-Sectional Studies

Study	Author (Year)	Country & Design	C1 Inclusion criteria defined	C2 Setting & subjects described	C3 Ethnicity measured validly	C4 Outcome measured validly	C5 Confounders identified	C6 Confounders controlled	C7 Appropriate statistics	C8 Sample size adequate	Overall Risk
**S1**	Afolabi et al. (2021)	Nigeria DHS 2018 GLMM	✓	✓	✓	✓	✓	✓	✓	✓	Low concern
**S2**	Masters et al. (2019)	Kenya DHS 2014 Multinomial LR	✓	✓	✓	✓	✓	✓	✓	✓	Low concern
**S3**	Ettarh et al. (2012)	Nairobi slum Surveillance Cox regression	✓	✓	✓	✓	✓	✓	✓	?	Low concern
**S4**	Adokiya et al. (2017)	Ghana Cluster survey Logistic LR	✓	✓	✓	✓	?	?	✓	✗	Moderate concern
**S5**	Egondi et al. (2015)	Kenya Slum survey Conc. index	✓	✓	✓	✓	✓	?	✓	✗	Moderate concern
**S6**	Antai (2011)	Nigeria DHS 2003 Multilevel LR	✓	✓	✓	✓	✓	✓	✓	✓	Low concern
**S7**	Adedini et al. (2015)	Nigeria DHS 2008 Cox regression	✓	✓	✓	✓	✓	✓	✓	✓	Low concern
**S8**	Ghose & Yaya (2020)	Nigeria MICS 2017 Multivariable LR	✓	✓	✓	✓	✓	✓	✓	✓	Low concern
**S9**	Fazzio et al. (2011)	Guinea-Bissau Retrospective KM + Cox	✓	✓	✓	✓	?	?	✓	✓	Moderate concern
**S10**	Tusting et al. (2024)	18 SSA countries 37 DHS surveys Logistic LR	✓	✓	✓	✓	✓	✓	✓	✓	Low concern

JBI Criteria for Analytical Cross-Sectional Studies: ✓ = Yes (criterion met); ✗ = No (criteria not met); ? = Unclear. Overall risk: Low = 7-8 criteria met; Moderate = 4-6 criteria met; High = 0-3 criteria unmet.
